# Antibody response to SARS-CoV-2 WT and Omicron BA.4/5 of inactivated COVID-19 vaccine in patients with lung cancer after second and booster immunization

**DOI:** 10.1186/s13045-023-01443-3

**Published:** 2023-05-03

**Authors:** Chen Chen, Liyuan Dai, Cuiling Zheng, Haolong Li, Xiaomeng Li, Mengwei Yang, Ruyun Gao, Jiarui Yao, Zhishang Zhang, Yuankai Shi, Xiaohong Han

**Affiliations:** 1 Clinical Pharmacology Research Center, Peking Union Medical College Hospital, State Key Laboratory of Complex Severe and Rare Diseases, NMPA Key Laboratory for Clinical Research and Evaluation of Drug, Beijing Key Laboratory of Clinical PK and PD Investigation for Innovative Drugs, Chinese Academy of Medical Sciences and Peking Union Medical College, No.1 Shuaifuyuan, Wangfujing, Dongcheng District, Beijing, 100730 China; 2 Department of Clinical Laboratory, National Cancer Center/National Clinical Research Center for Cancer/Cancer Hospital, Chinese Academy of Medical Sciences and Peking Union Medical College, No. 17 Panjiayuan Nanli, Chaoyang District, Beijing, 100021 China; 3 Department of Clinical Laboratory, State Key Laboratory of Complex, Severe and Rare Diseases, Peking Union Medical College Hospital, Chinese Academy of Medical Science and Peking Union Medical College, Beijing, 100730 China; 4 Department of Medical Research Center, Peking Union Medical College Hospital, Chinese Academy of Medical Science and Peking Union Medical College, Beijing, 100730 China; 5 Department of Medical Oncology, National Cancer Center/National Clinical Research Center for Cancer/Cancer Hospital, Chinese Academy of Medical Sciences and Peking Union Medical College, Beijing Key Laboratory of Clinical Study on Anticancer Molecular Targeted Drugs, No. 17 Panjiayuan Nanli, Chaoyang District, Beijing, 100021 China

**Keywords:** Lung cancer, SARS-CoV-2, Inactivated vaccine, Antibody response, Omicron variant

## Abstract

COVID-19 inactivated vaccine-induced humoral responses in patients with lung cancer (LCs) to SARS-CoV-2 wild-type (WT) strain and variants BA.4/5 after the primary 2-dose and booster vaccination remained unknown. We conducted a cross-sectional study in 260 LCs, 140 healthy controls (HC) and additional 40 LCs with serial samples by detecting total antibodies, IgG anti-RBD and neutralizing antibodies (NAb) toward WT and BA.4/5. SARS-CoV-2-specific antibody responses were augmented by the booster dose of inactivated vaccines in LCs, whereas they were lower than that in HCs. Enhanced humoral responses waned over time after triple injection, notably in NAb against WT and BA.4/5. The NAb against BA.4/5 was much lower than WT. Age ≥ 65 was risk factor for immunization of NAb to WT. Undergoing treatment resulted in a lower antibody response than those without and radiotherapy was a also risk factor for seroconversion of NAb to WT. Lower lymphocyte counts contributed to a lower titer of IgG anti-RBD and NAb against BA.4/5 in LCs than HCs. Specifically, total B cells, CD4^+^T cells and CD8^+^T counts were correlated with the humoral response. These results should be taken into consideration for the elderly patients under treatment.

**Supplementary Information:**

The online version contains supplementary material available at 10.1186/s13045-023-01443-3.

## To the Editor,

Since SARS-CoV-2 spread all over the world, patients with lung cancer (LCs) had an estimated case fatality rate of more than 30%, compared with 0.7% to 8.0% in general population [1]. As the median age of LCs diagnosis was 70 years, and immune dysregulation because of the need to receive anticancer therapy for the remainder of their lives [2, 3], antibody responses to two-dose inactivated vaccine in LCs were low [4]. In addition, blunted humoral responses to two-dose and booster mRNA vaccination were found in LCs [5, 6]. However, scarcely anything was known about the magnitude, quantity and duration of antibody response of booster dose of inactivated vaccine in LCs.

In addition, the Omicron variant harboring 30–40 mutations in the viral spike protein produced high immune evasion [7]. Homologous inactivated vaccine BBIBP-CorV booster improved neutralizing activity against Omicron variant in general population [8]. However, inactivated vaccine-induced immune responses to current predominant variants BA.5 in LCs remain unknown.

**Fig. 1:**
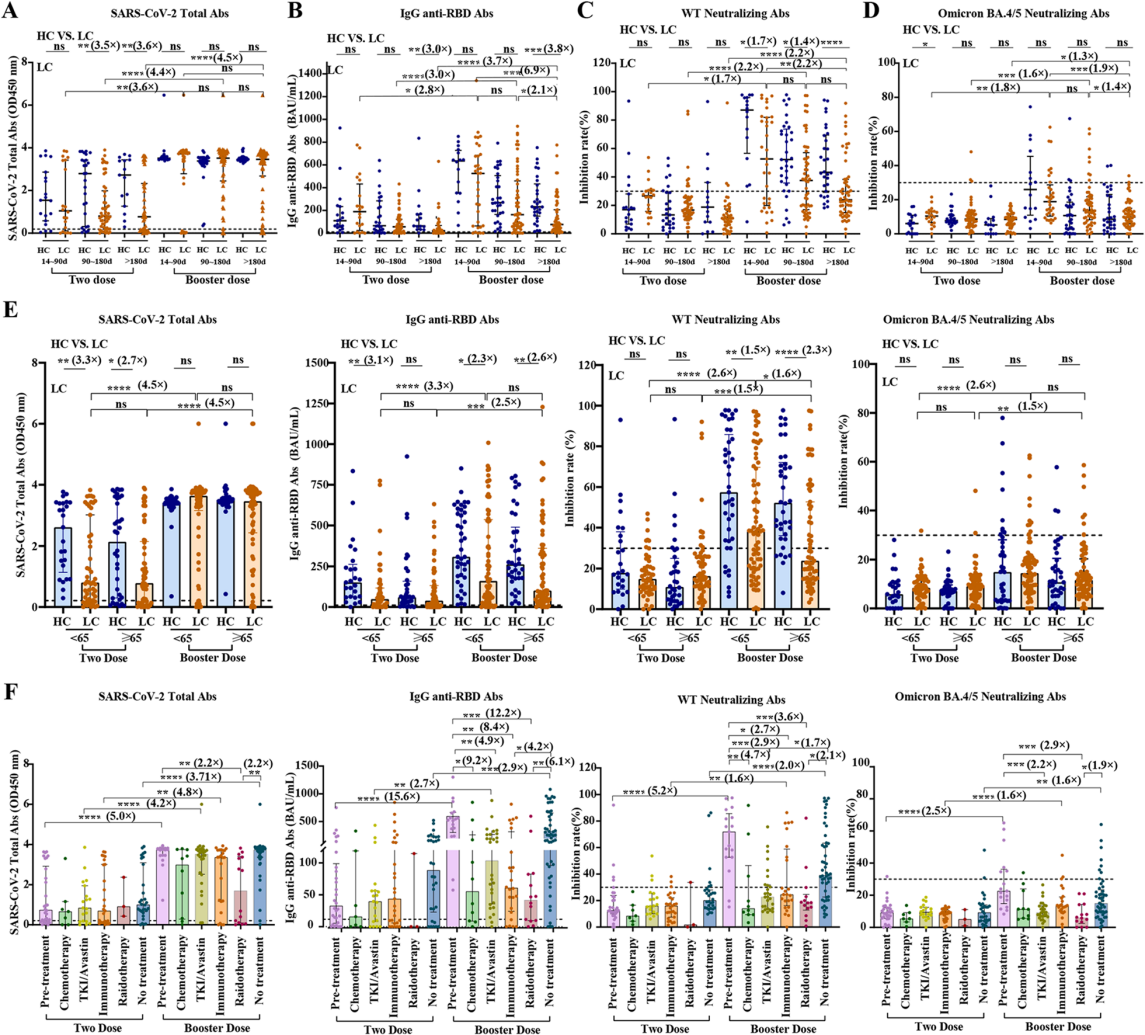
Inactivated COVID-19 vaccination-induced antibody responses and Omicron neutralization in patients with lung cancer. **A** Levels of total antibodies against SARS-CoV-2 in LCs and HCs after the second and booster dose of inactivated vaccine. **B** Concentrations (BAU/mL) of IgG anti-RBD antibodies in LCs and HC subjects after the second and booster dose of inactivated vaccine. **C** Inhibition rates (%) of NAb against WT evaluated by SARS-CoV-2 surrogate virus neutralization test (sVNT) in LCs and HC subjects after the second and booster dose of inactivated vaccine. **D** Inhibition rates (%) of NAb against Omicron BA.4/5 evaluated by SARS-CoV-2 surrogate virus neutralization test (sVNT) in LCs and HC subjects after the second and booster dose of inactivated vaccine. **E** Correlative analyses between SARS-CoV-2-specific antibody and patient’s age: two-dose and booster vaccination. **F** Correlative analyses between SARS-CoV-2-specific antibody and patient’s therapy: two-dose and booster vaccination

To address these key issues, we studied the humoral responses to inactivated vaccines in 260 LCs (Table 1), 140 age, sex and vaccination period matched healthy controls (HCs) after the second and booster vaccines (Additional file 1: Methods, Additional file 2: Table S1). Total antibodies against SARS-CoV-2 were 3.5-fold higher in HCs than LCs after the second dose. It showed > threefold increment post-booster vaccine (*P* < 0.05) than the second in LCs. A durability of total antibodies was found (14–90 days vs. > 180 days, *P* = 0.1890) (Fig. 1A). IgG anti-SARS-CoV-2 spike RBD antibodies results supporting increased response post-booster shot with a 2.8-fold increment in 14–90 days (*P* = 0.0422) in LCs, but it decreased faster than HCs and the result showed it was 3.8-fold higher in HCs than LCs after 180 days post-booster vaccine (*P* = 0.0003) (Fig. 1B). We further examined the neutralizing antibody (NAb) against the SARS-CoV-2 WT and Omicron. Overall, booster recipients exhibited increased NAb to WT in LCs, although it was lower than HCs. The waning tendency of NAb against WT and BA.4/5 post-third dose was found in both LCs and HCs (Fig. 1C, D; Additional file 2: Table S2), which had also been confirmed in other 40 LCs with serial samples (Additional file 2: Table S3, Additional file 3: Fig. S1). By comparison, ancestral SARS-CoV-2 neutralization ability was 1.3–2.8-fold higher compared with Omicron (*P* < 0.05) in LCs (Additional file 4: Fig. S2 and Additional file 2: Table S4).

**Table 1:** Demographics and clinical characterization of 260 patients with LC

Parameter	2nd dose after 14–89 days *n* (%)	2nd dose after 90–180 days *n* (%)	2nd dose after 180 days *n* (%)	3rd dose after 14–89 days *n* (%)	3rd dose after 90–180 days *n* (%)	3rd dose after 180 days *n* (%)
Number	20	54	42	33	56	55
Age						
Year^a^	69.30 ± 7.160	65.5 [61–68]	64 [59–67]	62.58 ± 10.90	63.5 [57–74]	66 [61–69]
< 65	6 (30)	22 (40.74)	23 (54.76)	17 (51.52)	31 (55.36)	25 (45.45)
≥ 65	14 (70)	32 (59.26)	19 (45.24)	16 (48.48)	25 (44.64)	30 (54.55)
Sex						
Female	5 (25)	16 (29.63)	14 (33.33)	10 (30.30)	19 (33.93)	21 (38.18)
Male	15 (75)	38 (70.37)	28 (66.67)	23 (69.70)	37 (66.07)	34 (61.82)
Histologic diagnosis						
NSCLC	19 (95)	53 (98.15)	38 (90.48)	28 (84.85)	53 (94.64)	45 (81.82)
SCLC	1 (5)	1 (1.85)	3 (7.14)	5 (15.15)	2 (3.57)	9 (16.36)
Others	0 (0)	0 (0)	1 (2.38)	0 (0)	0 (0)	1 (1.82)
Unknown	0 (0)	0 (0)	0 (0)	0 (0)	1 (1.79)	0 (0)
Stage						
I/II	7 (35)	12 (22.22)	10 (23.81)	12 (36.36)	24 (42.86)	15 (27.27)
III/IV	9 (45)	33 (61.11)	27 (64.29)	11 (33.33)	22 (39.29)	25 (45.45)
Unknown	4 (20)	9 (16.67)	5 (11.90)	10 (30.30)	10 (17.86)	15 (27.27)
Last treatment received < 3 mo						
Pre-treatment	4 (20)	12 (22.22)	14 (33.33)	10 (30.30)	8 (14.29)	0 (0)
Chemotherapy	1 (5)	3 (5.56)	3 (7.14)	0 (0)	2 (3.57)	8 (14.55)
Oral TKI or bevacizumab	2 (10)	11 (20.37)	6 (14.29)	3 (9.09)	5 (8.93)	20 (36.36)
Immunotherapy	4 (20)	11 (20.37)	12 (28.57)	4 (12.12)	11 (19.64)	9 (16.6)
Radiotherapy	0 (0)	1 (1.85)	2 (4.76)	5 (15.15)	4 (7.14)	5 (9.09)
No systemic treatment	9 (45)	16 (29.63)	5 (11.90)	10 (30.30)	25 (44.64)	13 (23.64)
Unknown	0 (0)	0 (0)	0 (0)	1 (3.03)	1 (1.79)	0 (0)
Clinical parameter						
WBC^a^	7.253 ± 2.188	5.94 [4.85–7.25]	6.312 ± 1.825	6.11 [5.215–6.895]	6.620 [5.290–8.145]	5.24 [3.74–6.93]
NEU^a^	5.020 ± 2.300	3.615 [2.960–4.580]	3.955 [3.220–5.140]	3.565 [3.150–4.575]	4.235 [3.235–5.460)	3.38 [2.210–4.340]
LYM^a^	1.670 [1.210–1.945]	1.716 ± 0.514	1.490 [1.180–1.880]	1.748 ± 0.540	1.645 [1.265–1.980]	1.260 [0.930–1.990]

LC, lung cancer; NSCLC, non-small cell lung cancer; SCLC, small cell lung cancer; TKI, tyrosine kinase inhibitor; WBC, white blood cell; NEU, neutrophil; and LYM, lymphocytes

^a^The data of these parameters was shown as mean ± SD or median (quartiles)

Next, we explored factors influenced the humoral response in these 260 LCs with single sampling and performed logistical analysis in 144 LCs received booster vaccines. Consisting with Ramasamy’s report in healthy people [9], the age did not influence expression of binding antibody in LCs (Fig. 1E). By contrast, in line with that NAb response to mRNA vaccination was age-dependent decline [2, 10], we did observe a statistically significant correlation between age and NAb toward WT in LCs and predictive value of LCs aged over 65 for lack of immunization (OR = 0.322, 95% CI 0.134–0.773, *P* = 0.0112) (Fig. 1E, Additional file 2: Table S5 and Table S7). This finding suggested that the elderly patients should be given more attention while planning vaccination programs.

Moreover, our results suggested undergoing various anticancer therapies influenced the antibody response of post-booster vaccine. LCs received radiotherapy generated lower level of total antibodies than other therapeutic strategies post-booster inactivated vaccines. The IgG anti-RBD antibodies titer, NAbs against WT and Omicron BA.4/5 were significantly lower in patients receiving various therapies than those without (Fig. 1F, Additional file 2: Table S6). Notably, the undergoing radiotherapy was an risk factor for immunization of NAb toward WT (OR = 0.082, 95% CI 0.011–0.617, *P* = 0.0151) in LCs (Additional file 2: Table S7). This reduced humoral response may be due to the immunosuppressive conditions and lymphocytes decrement induced by chemotherapy or radiotherapy [11]. Further research revealed that the lymphocyte counts were indeed significant lower in the LCs (1.56[1.213–2.018]) than HC (1.76[1.428–2.198]) (*P* = 0.0017) (Additional file 5: Fig. S3). In addition, the lymphocyte counts showed a positive correlation with the IgG anti-RBD antibodies (*P* = 0.0266) and Omicron BA.4/5 in LCs (*P* = 0.0339). The lower immunization may be explained by lower number total B cells, CD4^+^T cells and CD8^+^T counts in LCs as their correlation to the humoral response (Additional file 6: Fig. S4).

Overall, our study revealed strengthened humoral responses post-booster vaccine among LCs, albeit lower than HCs. However, the booster dose failed to establish a potent and durable antibody response for Omicron BA.4/5, which gives rise to the risk of breakthrough infections of Omicron variants, especially in those old and undergoing anticancer therapies. Given the lower antibodies in LCs receiving various active anticancer therapies, further studies are needed to determine whether increased dosage, mixing vaccine types or additional doses enhance immunogenicity.

## Supplementary Information

**Additional file 1:** Methods.

**Additional file 2: Table S1.** Matched demographics between 260 patients with LC and 140 HCs. **Table S2.** Antibody Response to SARS-CoV-2 inactivated Vaccine between 260 patients with LC and 140 HCs. **Table S3.** Demographics and clinical characterization of 40 patients with LC with sequential samples. **Table S4.** Comparative analysis of neutralizing effect responses to SARS-CoV-2 WT and Omicron variant BA.4/5 in 260 patients with LC. **Table S5.** Antibody response to SARS-CoV-2 inactivated vaccination in 260 LCs and 140 HCs aged < 65 and ≥ 65 years. **Table S6.** Antibody response to SARS-CoV-2 inactivated vaccination in 260 LCs receiving various treatment regimens. **Table S7.** Risk factors associated with seropositivity of SARS-CoV-2 antibodies in 144 LCs received booster vaccine.

**Additional file 3: Figure S1.** SARS-CoV-2 antibodies response in 40 LC patients after the second or booster dose of inactivated vaccine. Total antibodies against SARS-CoV-2. Concentrations of IgG anti-RBD antibodies. Inhibition rates of NAb against SARS-CoV-2 WT. Inhibition rates of NAb against Omicron BA.4/5.

**Additional file 4: Figure S2.** Comparison of neutralizing effect responses to SARS-CoV-2 WT and Omicron variant BA.4/5 in LCs. The figures show the median and quartiles. *P < 0.05, **P < 0.01, ***P < 0.001 and ****P < 0.0001. LC, lung cancer; WT, wild type.

**Additional file 5: Figure S3.** Comparison of lymphocytes counts in 260 LCs and HCs. The figures show the median and quartiles. **P < 0.01.

**Additional file 6: Figure S4.** Correlation of biological variables and magnitude of SARS-CoV-2 antibodies after the booster dose.

## Data Availability

Data are available upon request by email to the correspondence author.

## Abbreviations

CI: Confidence interval
COVID-19: Coronavirus disease 2019
HC: Healthy control
HRP: Horseradish peroxidase
IQR: Interquartile range
LC: Lung cancer
LYM: Lymphocytes
NAb: Neutralizing antibodies
NC: Negative control
NEU: Neutrophil
NSCLC: Non-small cell lung cancer
OD: Optical density
OR: Odds ratio
PC: Positive control
RBD: Receptor-binding domain
SARS-CoV-2: Severe acute respiratory syndrome coronavirus 2
SD: Standard deviation
sVNT: Surrogate virus neutralization test
TKI: Tyrosine kinase inhibitor
TMB: Tetramethyl benzidine
WBC: White blood cell
