# Supplementary material for: Antibody response to SARS-CoV-2 WT and Omicron BA.4/5 of inactivated COVID-19 vaccine in patients with lung cancer after second and booster immunization
Source: J Hematol Oncol. 2023 May 3;16:47. doi: 10.1186/s13045-023-01443-3 (PMC10155141; doi:10.1186/s13045-023-01443-3)
Supplement: Supplementary file 1 — Additional file 1: Methods. [file 13045_2023_1443_MOESM1_ESM.docx]

**Materials and Methods**

**Lung cancer patients and specimen collection**


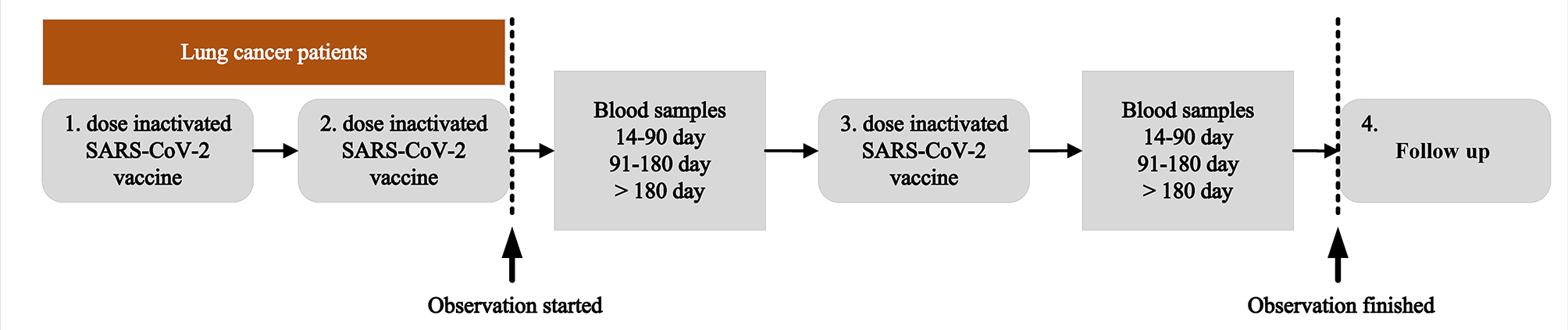
A total of 260 peripheral blood samples from 260 LCs, 140 age and sex, and vaccination period matched healthy controls (HCs), and 82 serial sera from the other 40 patients with LC vaccinated with inactive COVID-19 vaccines were collected from July 2021 to September 2022 in Cancer Hospital Chinese Academy of Medical Sciences and Peking Union Medical College Hospital. Inclusion criteria of LC patients were as follows: (1) adult patients’ diagnosis were confirmed by biopsy; (2) received two or third dose regimen of inactivated SARS-CoV-2 vaccine; (3) with detailed clinical data and laboratory parameters, including age, histopathology types, therapy, white blood cells (WBC), neutrophiles (NEU), and lymphocytes (LYM). Patients with SARS-CoV-2 infection history were excluded from this study (as showed in the diagram).

All patients with LC were categorized into six subgroups: 14-90 days (n=20), 91-180 days (n=54) and >180 days (n=42) after receiving the second dose of inactivated vaccine, and the same period after booster dose 14-90 days (n=33), 91-180 days(n=56) and >180 days (n=55). On the basis of the type of therapy received within 3 months before the blood sampling time, the LC cohort was divided into six subsets to vaccination: patients were at pretherapy, patients receiving chemotherapy (chemotherapy alone), oral tyrosine kinase inhibitor therapy (TKI) / Avastin (oral TKI, Avastin + chemotherapy), immunotherapy (immunotherapy alone, ICI + chemotherapy, ICI + Avastin, ICI + oral TKI, ICI + Avastin + chemotherapy), radiotherapy (radiotherapy alone, radiotherapy + chemotherapy, radiotherapy + oral TKI, radiotherapy + ICI + chemotherapy), those without systemic therapy (surgery) or follow up within the past 3 months at sampling time.

Supernatant were collected and stored in 1.5 mL tubes at -80°C after peripheral blood being centrifuged at 3000 rpm and 4 °C for 10 minutes, and supernatant were thawed until used. All samples were collected after routine clinical tests. For laboratory tests, White blood cell counts were measured using Sysmex XN-1000 Pure. B cell, NK cell, CD4^+^ and CD8 T^+^ lymphocytes were analyzed by Flow CytoMetry on the BD FACS Calibur in 69 patients with LC with booster vaccines. Electronic medical records of LC patients including age, histopathology types, therapy, WBC, NEU, and LYM were retrieved.

This study has been approved by the Ethics Committee of the National Cancer Center/National Clinical Research Center for Cancer/Cancer Hospital, Peking Union Medical College and the Chinese Academy of Medical Sciences Permission (22/363-3565) and Peking Union Medical College Hospital (I-22PJ354). All experiments were performed according to the Declaration of Helsinki.

**Total antibodies against SARS-CoV-2 assay**

The total antibodies (including IgM and IgG) against SARS-CoV-2 RBD region of S1 subunit (antigen) was detected using the kit purchased from the Beijing Wantai Biological Pharmacy Enterprise, Beijing, China. The experiment was performed under the instruction of the manufacturer as previous reports ^1^. Briefly, a total of 100 μL of samples or 50 uL positive/negative controls (PCs/NCs) were added into a microwell plate coated with recombinant SARS-CoV-2 antigen RBD region of S1 subunit and incubated at 37°C for 30 min. After five cycles of wash, 100 uL horseradish peroxidase (HRP)- labeled 2019-nCoV-Ag was added. After washing, 50 μL of chromogenic agents A (≥ 0.3 g/L peroxide) and B (≥ 0.2 g/L HRP) was added sequentially. Finally, added with 50 μL of stop solution. The optical density (OD) at 450 nm were measured using Multiskan GO automatic microplate reader (Thermo, USA). OD value = Raw OD – Blank OD. 0.19 was calculated was the cut off value to divide seropositive and seronegative samples in the present study using the formula provided by the manufacture: cut off value = 0.16+ mean OD value of negative control well.

**IgG anti-SARS-CoV-2 spike RBD antibody assay**

The IgG anti-SARS-CoV-2 spike RBD was detected using the kit purchased from PROPRIUM Co., Ltd. According to the manufacturer's instruction as previous reports ^1^, 100 uL calibrators or plasma samples diluted in 1:300-fold were added to the microplates which were pre-coated with the recombinant SARS-CoV-2 RBD antigen. After washing, 100 uL anti-human IgG-HRP antibody (1:3000) was added to incubate for 30 min. Then the substrate Tetramethyl benzidine (TMB) was added and after incubated for 10 minutes, the reaction was stopped with 50 uL stop solution. OD (450 nm) were measured. The concentration (BAU/mL) of anti-RBD antibodies was calculated by the standard curves with a reference range of 10–1000 BAU/mL. 11.6 BAU/ml was a threshold to divide sero-positive and -negative samples provided by the manufacture.

**Neutralizing antibody against SARS-CoV-2 wild type (WT) and Omicron BA.4/5 assay**

The SARS-CoV-2 Surrogate Virus Neutralization Test (sVNT) kit (SARS-CoV-2 Neutralization Antibody Detection Kit, Genscript) was employed to detect the neutralizing antibodies that block the interaction between the viral RBD protein and human ACE2 (hACE2) ^2^. The sVNT kit was highly correlated with both cVNT and pVNT and it has been validated in two COVID-19 patient cohorts in two different countries with 100% SP and 95-100% SE. sVNT was conducted for serum IgG anti-SARS-CoV-2 WT and Omicron BA.4/5 antibodies.

According to the manufacturer's instruction as previous reports ^1^, samples and PCs/NCs were 1:10 diluted with the provided reagent and mixed with the same volume of HRP-RBD, which were incubated at 37°C for 30 minutes. Then 100 μl of the mixture was added to the microwell strips 37°C for 15 minutes. After washing, 100 μl TMB was added to each well. And 15 minutes later, 50 uL stop solution was added. Then OD (450 nm) was measured. The inhibition rate (%) was calculated as follows: Inhibition rate (%) = (1–OD value of sample/OD value of NC) * 100%. According to the manufacture, the inhibition rate ≥30% was regarded as positive in SARS-CoV-2-neutralizing antibody.

**Statistical analysis**

Descriptive statistics were performed to tabulate patients’ demographic and clinical characteristics. For continuous variables, the normality of data distribution was assessed using the Shapiro–Wilk test. Normally distributed data were reported as mean ± standard deviation (SD), while non-normally distributed data were presented as median [IQR]; Mann-Whitney test for continuous variables was used when comparing two independent groups with non-normal distributions. Statistical differences between multiple groups were assessed by Kruskal-Wallis test and multiple comparison was accounted for by using Dunn’s test. Chi-square or Fischer’s exact test were used for categorical variables of the positivity rate of the total antibodies against SARS-CoV-2, IgG anti-RBD antibodies, NAb against WT, and NAb against BA.4/5. The strength of the association between SARS-CoV-2 antibodies and laboratory biomarkers was tested with the Spearman test and the coefficient values >0.3 or <−0.3 considered clinically relevant. Further multivariate logistic regression analyses were conducted to assess independent risk factors for the positivity of the above-mentioned antibodies after the third dose of the inactivated vaccine among patients with LC. In multivariable analysis, age, sex, time period after the booster vaccine, histologic diagnosis, stage, types of treatment, WBC, NEU, LYM were considered, except for lymphocytes subset of NK cells, B cells, CD4^+^T-cell counts and CD4^+^T-cell counts because of their small sample size. For all analyses, two-tailed *P* < 0.05 was considered statistically significant. The statistical analysis was conducted using GraphPad Prism 9 (San Diego, CA, USA), SAS Version 9.4 (SAS Institute Inc., Cary, NC, USA) and R version 4.2.1 software.

Reference

1. Zhan H, Gao H, Liu Y, et al. Booster shot of inactivated SARS-CoV-2 vaccine induces potent immune responses in people living with HIV. J Med Virol*.* 2023;95:e28428.

2. Terpos E, Zagouri F, Liontos M, et al. Low titers of SARS-CoV-2 neutralizing antibodies after first vaccination dose in cancer patients receiving checkpoint inhibitors. J Hematol Oncol*.* 2021;14:86.
