# Supplementary material for: Antibody response to SARS-CoV-2 WT and Omicron BA.4/5 of inactivated COVID-19 vaccine in patients with lung cancer after second and booster immunization
Source: J Hematol Oncol. 2023 May 3;16:47. doi: 10.1186/s13045-023-01443-3 (PMC10155141; doi:10.1186/s13045-023-01443-3)
Supplement: Supplementary file 2 — Additional file 2: Table S1. Matched demographics between 260 patients with LC and 140 HCs. Table S2. Antibody Response to SARS-CoV-2 inactivated Vaccine between 260 patients with LC and 140 HCs. Table S3. Demographics and clinical characterization of 40 patients with LC with sequential samples. Table S4. Comparative analysis of neutralizing effect responses to SARS-CoV-2 WT and Omicron variant BA.4/5 in 260 patients with LC. Table S5. Antibody response to SARS-CoV-2 inactivated vaccination in 260 LCs and 140 HCs aged < 65 and ≥ 65 years. Table S6. Antibody response to SARS-CoV-2 inactivated vaccination in 260 LCs receiving various treatment regimens. Table S7. Risk factors associated with seropositivity of SARS-CoV-2 antibodies in 144 LCs received booster vaccine. [file 13045_2023_1443_MOESM2_ESM.docx]

Supplementary Table 1. Matched demographics between 260 patients with LC and 140 HCs.

| Period time of sampling | Group | Number | Age (year) | Gender (F/M) | Time of sampling (day) |
| --- | --- | --- | --- | --- | --- |
| 2nd dose after 14-89 days | LC | 20 | 69.30±7.160 | 5/15 | 54.10±23.06 |
|  | HC | 19 | 68.53±8.847 | 5/14 | 52.42±29.58 |
|  | ***P*** | | 0.7652 | 0.9251 | 0.8439 |
| 2nd dose after 90-180 days | LC | 54 | 65.5[61.0-68.0] | 16/38 | 142[122-163] |
|  | HC | 27 | 66[60-74] | 9/18 | 134.6±23.37 |
|  | ***P*** | | 0.7366 | 0.7337 | 0.3187 |
| 2nd dose after 180 days | LC | 42 | 64[59-67] | 14/28 | 219.7±20.48 |
|  | HC | 15 | 60[40-66] | 9/6 | 190[185-222] |
|  | ***P*** | | 0.1794 | 0.0708 | 0.0558 |
| 3rd dose after 14-89 days | LC | 33 | 62.58±10.90 | 10/23 | 61[29-75] |
|  | HC | 16 | 59.87±8.293 | 5/11 | 70[59-83] |
|  | ***P*** | | 0.3866 | 0.9426 | 0.1050 |
| 3rd dose after 90-180 days | LC | 56 | 63.5[57-74] | 19/37 | 136.9±24.27 |
|  | HC | 33 | 64[61-67] | 11/22 | 140.0±20.08 |
|  | ***P*** | | 0.5890 | 0.9542 | 0.5396 |
| 3rd dose after 180 days | LC | 55 | 66[61-69] | 21/34 | 228[210-256] |
|  | HC | 30 | 67[63-69] | 10/20 | 227.3±26.26 |
|  | ***P*** | | 0.7228 | 0.6572 | 0.8181 |

LC: lung cancer; HC, healthy control; F, female; M, male.

Supplementary Table 2. Antibody Response to SARS-CoV-2 inactivated Vaccine between 260 patients with LC and 140 HCs.

| Antibody | Group | 2nd dose after 14-90 days | 2nd dose after 91-180 days | 2nd dose after 180 days | 3rd dose after 14-90 days | 3rd dose after 91-180 days | 3rd dose after 180 days |
| --- | --- | --- | --- | --- | --- | --- | --- |
| SARS-CoV-2 total antibodies (OD value) | LC | 1.048[0.1123-3.425] | 0.7991[0.2684-1.987] | 0.7669[0.1388-2.329] | 3.721[2.786-3.816] | 3.515[2.444-3.793] | 3.461[2.681-3.75] |
|  | HC | 1.738±1.317 | 2.794[0.3128-3.423] | 2.727[1.264-3.438] | 3.511[3.462-3.626] | 3.383[3.252-3.534] | 3.454[3.399-3.545] |
|  | ***P*** | 0.4440 | 0.0084 | 0.0062 | 0.5938 | 0.1394 | 0.7856 |
| IgG anti-RBD antibody (BAU/mL) | LC | 188.7[23.59-431.8] | 55.34[12.8-122.4] | 20.97[0-67.71] | 524.7[63.56-684.2] | 162.5[58.86-458.8] | 76.53[29.99-206.2] |
|  | HC | 109.7[49.97-233.9] | 65.19[25-284] | 63.47[32.22-169.4] | 541.3±234.6 | 316.8±210.6 | 288.1±200.2 |
|  | ***P*** | 0.4607 | 0.1365 | 0.0024 | 0.2147 | 0.1155 | 0.0003 |
| NAb towards WT (Inhibition%) | LC | 26.68[15.79-30.56] | 16.73[12.49-24.98] | 10.88[5.988-17.48] | 52.65[19.91-81.88] | 37.51[17-57.04] | 23.87[14.95-38.25] |
|  | HC | 17[6.867-28.04] | 13.31[6.222-29.09] | 18.79[8.235-36.12] | 87.03[56.59-95.8] | 54±25.73 | 50.14±23.27 |
|  | ***P*** | 0.0612 | 0.1725 | 0.1231 | 0.0118 | 0.0158 | <0.0001 |
| NAb towards BA.4/5 (Inhibition%) | LC | 10.32±5.185 | 8.602[5.143-12.39] | 7.822±4.748 | 18.82[10.32-28.65] | 13.62[8.236-21.67] | 9.856[5.859-15] |
|  | HC | 6.011[0-10.9] | 7.753[6.611-10.9] | 5.221[0-8.912] | 28.97±21.5 | 10.71[2.685-18.53] | 8.561[3.419-23.04] |
|  | ***P*** | 0.0161 | 0.7996 | 0.0775 | 0.3046 | 0.0537 | 0.7751 |

Abbreviations: LC, lung cancer; HC, healthy control; OD, optimal density; RBD, receptor binding domain; WT, wild type; NAb, neutralizing antibody.

Supplementary Table 3. Demographics and clinical characterization of 40 patients with LC with serial samples.

| Parameter | LC |
| --- | --- |
| Number | 40 |
| Age | |
| Median(range) | 66(42-74) |
| <65 | 18 |
| ≥65 | 22 |
| SEX | |
| Female | 8 |
| Male | 32 |
| Histologic diagnosis | |
| NSCLC | 36 |
| SCLC | 4 |
| Stage |  |
| I+II | 3 |
| III+IV | 33 |
| Unknown | 4 |
| Last treatment received <3 mo | |
| Pre-treatment | 23 (25 samples) |
| Chemotherapy | 1 (1 sample) |
| Oral TKI or bevacizumab | 3 (4 sample) |
| Immunotherapy | 32 (49 samples) |
| Radiotherapy | 1 (1 sample) |
| No systemic treatment | 1 (1 sample) |
| Unknown | 1 (1 sample) |
| Clinical parameter | |
| WBC ^1^ | 7.06±2.57 |
| NEU ^1^ | 4.57±2.15 |
| LYM ^1^ | 1.84±0.70 |

Abbreviations: LC, lung cancer; NSCLC, non-small cell lung cancer; SCLC, small cell lung cancer; TKI, tyrosine kinase inhibitor; WBC, white blood cell; NEU, neutrophil; LYM, lymphocytes.

^1^ The data of these parameters was shown as mean±SD.

Supplementary Table 4. Comparative analysis of neutralizing effect responses to SARS-CoV-2 WT and Omicron variant BA.4/5 in 260 patients with LC.

| Sampling time | WT | BA.4/5 | Fold change | ***P*** |
| --- | --- | --- | --- | --- |
| Second dose after 14-90 days | 26.68[15.79-30.56] | 10.32±5.185 | 2.570327553 | <0.0001 |
| Second dose after 91-180 days | 16.73[12.49-24.98] | 8.602[5.143-12.39] | 1.944896536 | <0.0001 |
| Second dose after 180 days | 10.88[5.988-17.48] | 7.822±4.748 | 1.28666036 | 0.0203 |
| Third dose after 14-90 days | 52.65[19.91-81.88] | 18.82[10.32-28.65] | 2.797555792 | <0.0001 |
| Third dose after 91-180 days | 37.51[17-57.04] | 13.62[8.236-21.67] | 2.754038179 | <0.0001 |
| Third dose after 180 days | 23.87[14.95-38.25] | 9.856[5.859-15] | 2.421875 | <0.0001 |

Abbreviations: LC, lung cancer; WT, wild type.

Supplementary Table 5. Antibody response to SARS-CoV-2 inactivated vaccination in 260 patients with LC and 140 HCs aged < 65 and ≥ 65 years.

| Group | | LC | | HC | |
| --- | --- | --- | --- | --- | --- |
| Antibodies | Age | Second dose | Boost dose | Second dose | Boost dose |
| SARS-CoV-2 total antibodies (OD value) | < 65 | 0.802[0.2205-3.012] | 3.638[3.154-3.793] | 2.608[1.137-3.408] | 3.421[3.325-3.525] |
|  | ≥ 65 | 0.7849[0.1453-2.149] | 3.458[2.429-3.752] | 2.139[0.2958-3.584] | 3.516[3.396-3.578] |
|  | ***P*** | 0.6327 | 0.4065 | 0.6849 | 0.0139 |
| IgG anti-RBD antibody (BAU/mL) | <65 | 49.19[5.04-116.9] | 160.4[49.59-535.7] | 151.7[61.5-263] | 368.1±227.2 |
|  | ≥ 65 | 39.64[6.526-132.1] | 101[46.79-361.1] | 60.82[27.12-159.1] | 262.8[177.2-489.7] |
|  | ***P*** | 0.9734 | 0.5012 | 0.3505 | 0.4491 |
| NAb towards WT (Inhibition%) | < 65 | 14.86[9.763-21.07] | 38.25[20-69.62] | 17.84[9.897-37.94] | 57.36[33.69-85.78] |
|  | ≥ 65 | 16.32[11.36-25.83] | 23.87[14.99-52.65] | 10.96[4.232-25] | 55.1±23.84 |
|  | ***P*** | 0.2327 | 0.0494 | 0.7294 | 0.5082 |
| NAb towards BA.4/5 (Inhibition%) | < 65 | 8.447[5.276-12.03] | 14.47[8.541-20.91] | 5.811[1.298-10.77] | 14.88[3.159-28.12] |
|  | ≥ 65 | 9.104[5.198-12.31] | 11.6[6.783-20.33] | 7.212[5.418-9.656] | 10.75[5.123-20.51] |
|  | ***P*** | 0.7775 | 0.155 | 0.1989 | 0.6289 |

Abbreviations: LC, lung cancer; HC, healthy control; OD, optimal density; RBD, receptor binding domain; WT, wild type; NAb, neutralizing antibody.

Number: LC: two dose: < 65, n=51; ≥ 65, n=65; LC: booster dose: < 65, n=73; ≥ 65, n=71; HC: two dose: < 65, n=25; ≥ 65, n=36; HC: booster dose: < 65, n=41; ≥ 65, n=38;

Supplementary Table 6. Antibody response to SARS-CoV-2 inactivated vaccination in 260 patients with LC receiving various treatment regimens.

| Antibodies | Vaccine time | Pre-treatment | Chemotherapy | TKI/Avastin | Immunotherapy | Radiotherapy | No treatment |
| --- | --- | --- | --- | --- | --- | --- | --- |
| SARS-CoV-2 total antibodies (OD value) | 2nd dose | 0.7407[0.1962-2.915] | 0.6631[0-1.158] | 0.8408[0.1669-1.945] | 0.6942[0.1153-3.006] | 1.235±1.008 | 1.006[0.6521-3.088] |
|  | 3rd dose | 3.74[3.454-3.815] | 2.99[0.2813-3.745] | 3.528[2.516-3.751] | 3.361[1.169-3.715] | 1.697[0.09425-3.406] | 3.733[3.369-3.826] |
|  | ***P*** ^a^ | < 0.0001 | 0.1047 | < 0.0001 | 0.0086 | 0.9529 | <0.0001 |
|  | ***P*** ^b^ | - | 0.3085 | > 0.9999 | 0.1582 | 0.0076 | > 0. 9999 |
| IgG anti-RBD antibody (BAU/ml) | 2nd dose | 32.85[5.618-98.94] | 15.69[0-118.9] | 39.64[8.1-56.34] | 43.74[5.967-126.3] | 0[0-114.7] | 88.57[22.98-221.4] |
|  | 3rd dose | 512.3±290.8 | 55.47[8.735-260.2] | 103.7[35.6-266.4] | 61[24.2-315.9] | 41.94[9.569-82.55] | 256.2[95.87-653.7] |
|  | ***P*** ^a^ | < 0.0001 | 0.3131 | 0.0075 | 0.3674 | 0.3618 | 0.0007 |
|  | ***P*** ^b^ | - | 0.0178 | 0.0085 | 0.0013 | 0.0002 | >0.9999 |
| NAb towards WT （Inhibition%） | 2nd dose | 12.75[9.907-23.82] | 11.12±8.459 | 16.04[10.07-25.42] | 15.75±9.701 | 11.84±18.98 | 20.13[14.44-27.99] |
|  | 3rd dose | 66.01±24.46 | 14.06[9.37-46.38] | 22.79[13.84-31.83] | 24.62[16.13-58.82] | 18.36[12.85-24.66] | 39.26[22.65-63.29] |
|  | ***P*** ^a^ | < 0.0001 | 0.1932 | 0.1068 | 0.0024 | 0.3618 | <0.0001 |
|  | ***P*** ^b^ | - | 0.0028 | 0.0003 | 0.0209 | 0.0002 | 0.5541 |
| NAb towards BA.4/5 (Inhibition%) | 2nd dose | 9.223[5.676-12.37] | 6.488±4.262 | 9.832±4.617 | 8.097±3.511 | 5.702±5.044 | 9.292[4.125-14] |
|  | 3rd dose | 22.64[14.79-36.18] | 11.31[5.622-23.94] | 10.11±5.817 | 13.35[9.813-18.95] | 7.895±7.133 | 15[9.313-23.38] |
|  | ***P*** ^a^ | < 0.0001 | 0.1331 | 0.8261 | < 0.0001 | 0.8603 | 0.0051 |
|  | ***P*** ^b^ | - | 0.1818 | 0.0003 | 0.2869 | 0.0002 | 0.3263 |

Abbreviations: LC, lung cancer; OD, optimal density; RBD, receptor binding domain; WT, wild type; NAb, neutralizing antibody.

***P*** ^a^, second dose vs. third dose vaccine in patients with various therapies;

***P*** ^b^, patients at pre-therapies vs. patients undergoing various therapies after the booster dose of vaccine.

Number: two dose: < 65, n=51;

Supplementary Table 7. Risk factors associated with seropositivity of SARS-CoV-2 antibodies in 144 patients with LC received booster vaccine.

| Parameter | IgG anti-RBD antibodies (125/19) ^a^ | | NAb against WT (72/72) ^a^ | | NAb against Omicron BA.4/5 (18/126) ^a^ | |
| --- | --- | --- | --- | --- | --- | --- |
|  | OR (95% CI) | ***P*** | OR (95% CI) | ***P*** | OR (95% CI) | ***P*** |
| Age | 0.2742 | | 0.0112 | | 0.6356 | |
| <65 | ref | | ref | | ref | |
| >=65 | 1.929 (0.594-6.261) | 0.2742 | 0.322 (0.134-0.773) | 0.0112 | 0.735 (0.206-2.627) | 0.6356 |
| Sex | 0.2969 | | 0.1479 | | 0.0201 | |
| Female | ref | | ref | | ref | |
| Male | 0.451 (0.101-2.012) | 0.2969 | 0.500 (0.196-1.278) | 0.1479 | 0.183 (0.044-0.766) | 0.0201 |
| Time | 0.5322 | | 0.3858 | | 0.3093 | |
| 14-90d after 3 doses | ref | | ref | | ref | |
| 91-180d after 3 dose | 1.857 (0.405-8.518) | 0.4257 | 0.715 (0.224-2.281) | 0.5713 | 0.590 (0.140-2.488) | 0.4719 |
| >180d after 3 dose | 2.476 (0.496-12.355) | 0.2691 | 0.428 (0.123-1.489) | 0.1821 | 0.157 (0.015-1.677) | 0.1255 |
| Histologic diagnosis | 0.8859 | | 0.9928 | | 0.9773 | |
| NSCLC | ref | | ref | | ref | |
| SCLC | 2.060 (0.353-12.021) | 0.4217 | 0.775 (0.148-4.045) | 0.7625 | 0.540 (0.037-7.954) | 0.6335 |
| Others | >999.999 (<0.001->999.999) | 0.9912 | >999.999 (<0.001->999.999) | 0.9894 | <0.001 (<0.001->999.999) | 0.9930 |
| unknown | >999.999 (<0.001->999.999) | 0.9914 | <0.001 (<0.001->999.999) | 0.9907 | 0.656 (<0.001->999.999) | 0.9997 |
| Stage | 0.2808 | | 0.8839 | | 0.2334 | |
| I/II | ref | | ref | | ref | |
| III/IV | 0.701 (0.074-6.606) | 0.7561 | 0.750 (0.173-3.248) | 0.7004 | 0.276 (0.029-2.592) | 0.2601 |
| Unknown | 0.236 (0.032-1.746) | 0.1575 | 0.711 (0.176-2.872) | 0.6323 | 2.238 (0.377-13.279) | 0.3751 |
| Therapy |  | 0.6376 | 0.0220 | | 0.5259 | |
| pre-treatment | >999.999 (<0.001->999.999) | 0.9628 | 4.52 (0.676-29.988) | 0.1200 | 7.920 (1.239-50.613) | 0.0288 |
| Chemotherapy | 0.215 (0.02-2.069) | 0.1833 | 0.348 (0.047-2.580) | 0.3019 | 5.942 (0.243-145.382) | 0.2747 |
| Oral TKI or bevacizumab | 1.605 (0.160-16.083) | 0.6874 | 0.338 (0.078-1.462) | 0.1465 | <0.001 (<0.001->999.999) | 0.9511 |
| Immunotherapy | 0.378 (0.038-3.773) | 0.4073 | 0.254 (0.044-1.541) | 0.1233 | 8.209 (0.467-144.426) | 0.1502 |
| radiotherapy | 0.221 (0.025-1.958) | 0.1751 | 0.082 (0.011-0.617) | 0.0151 | <0.001 (<0.001->999.999) | 0.9655 |
| No systemic treatment | ref | | ref | | ref | |
| unknown | >999.999 (<0.001->999.999) | 0.9872 | <0.001 (<0.001->999.999) | 0.9855 | <0.001 (<0.001->999.999) | 0.9870 |
| Clinical parameter | | | | | | |
| WBC | 0.348 (0.073-1.649) | 0.1835 | 0.739 (0.142-3.856) | 0.7197 | 1.248 (0.255-6.102) | 0.7842 |
| NEU | 2.795 (0.558-14.011) | 0.2114 | 1.237 (0.218-7.009) | 0.8104 | 0.770 (0.146-4.069) | 0.7579 |
| LYM | 7.425 (0.824-66.943) | 0.0739 | 1.731 (0.262-11.455) | 0.5690 | 0.893 (0.109-7.338) | 0.9159 |

Abbreviations: LC, lung cancer; WBC, white blood cell; NEU, neutrophil; LYM, lymphocytes.

^a^ The number of positive/negative samples for each antibody assay.
