# Supplementary figures and images for: Antibody response to SARS-CoV-2 WT and Omicron BA.4/5 of inactivated COVID-19 vaccine in patients with lung cancer after second and booster immunization
Source: J Hematol Oncol. 2023 May 3;16:47. doi: 10.1186/s13045-023-01443-3 (PMC10155141; doi:10.1186/s13045-023-01443-3)

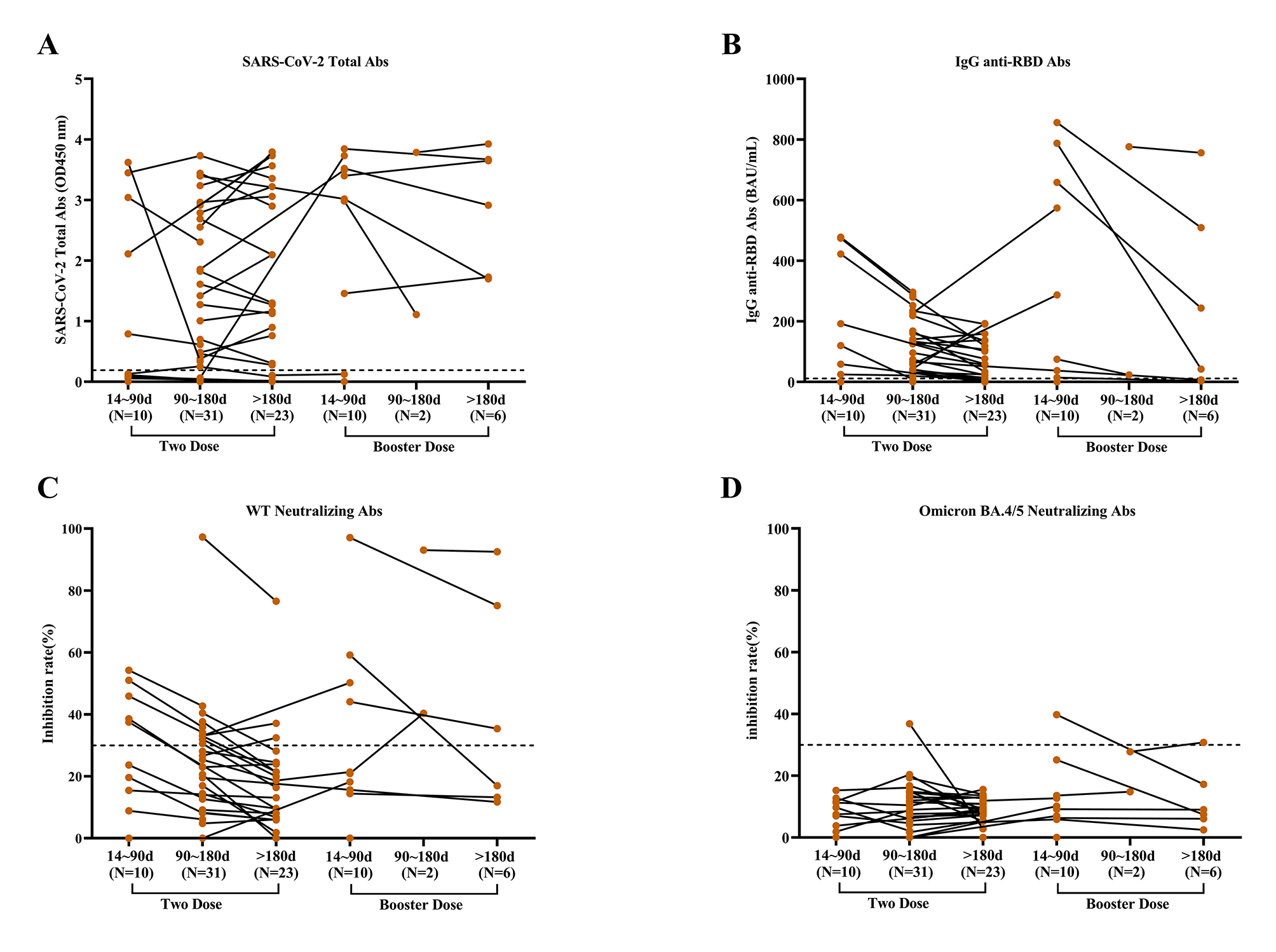

Supplement: Supplementary file 3 — Additional file 3: Figure S1. SARS-CoV-2 antibodies response in 40 LC patients after the second or booster dose of inactivated vaccine. Total antibodies against SARS-CoV-2. Concentrations of IgG anti-RBD antibodies. Inhibition rates of NAb against SARS-CoV-2 WT. Inhibition rates of NAb against Omicron BA.4/5. [file 13045_2023_1443_MOESM3_ESM.tif]

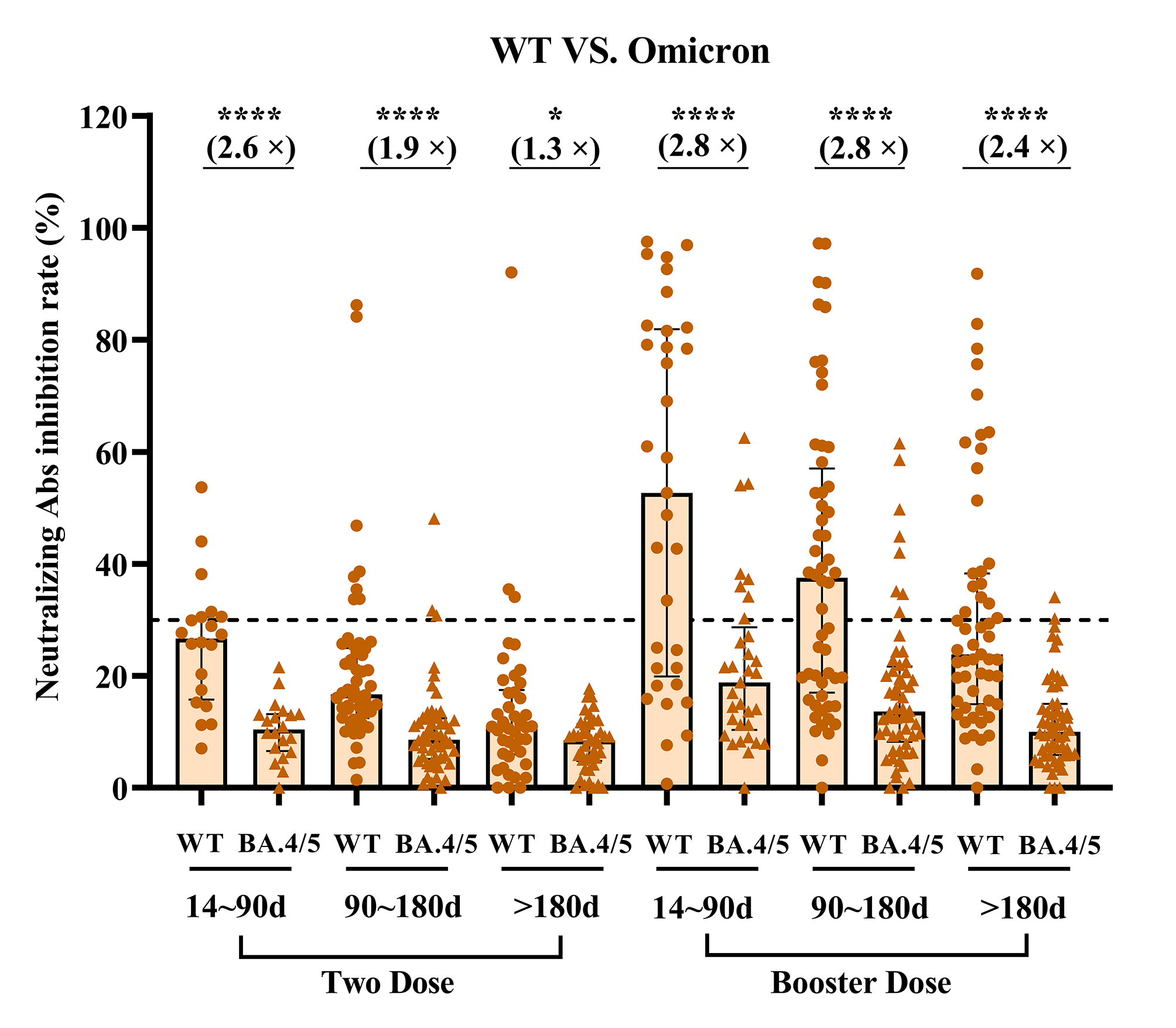

Supplement: Supplementary file 4 — Additional file 4: Figure S2. Comparison of neutralizing effect responses to SARS-CoV-2 WT and Omicron variant BA.4/5 in LCs. The figures show the median and quartiles. *P < 0.05, **P < 0.01, ***P < 0.001 and ****P < 0.0001. LC, lung cancer; WT, wild type. [file 13045_2023_1443_MOESM4_ESM.tif]

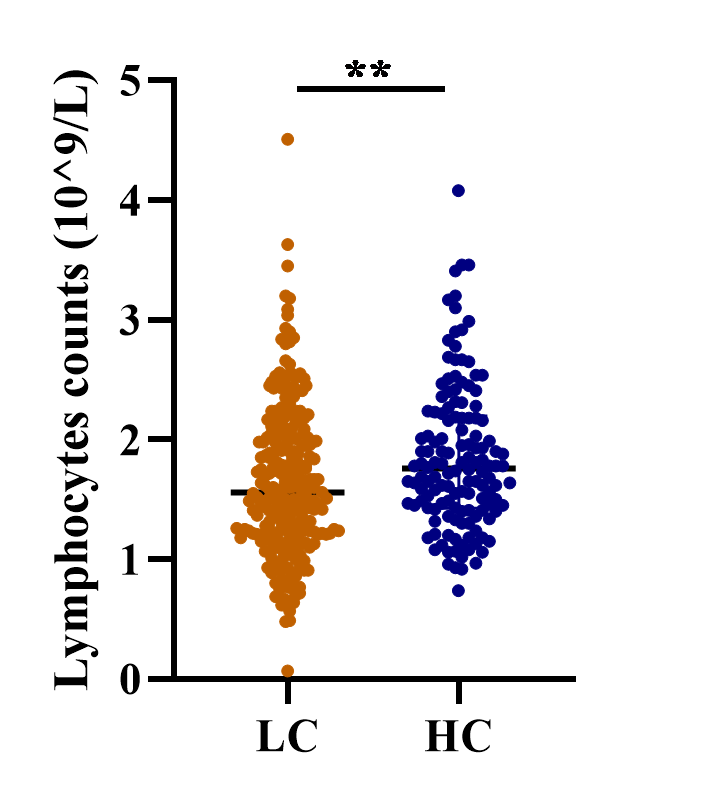

Supplement: Supplementary file 5 — Additional file 5: Figure S3. Comparison of lymphocytes counts in 260 LCs and HCs. The figures show the median and quartiles. **P < 0.01. [file 13045_2023_1443_MOESM5_ESM.tif]

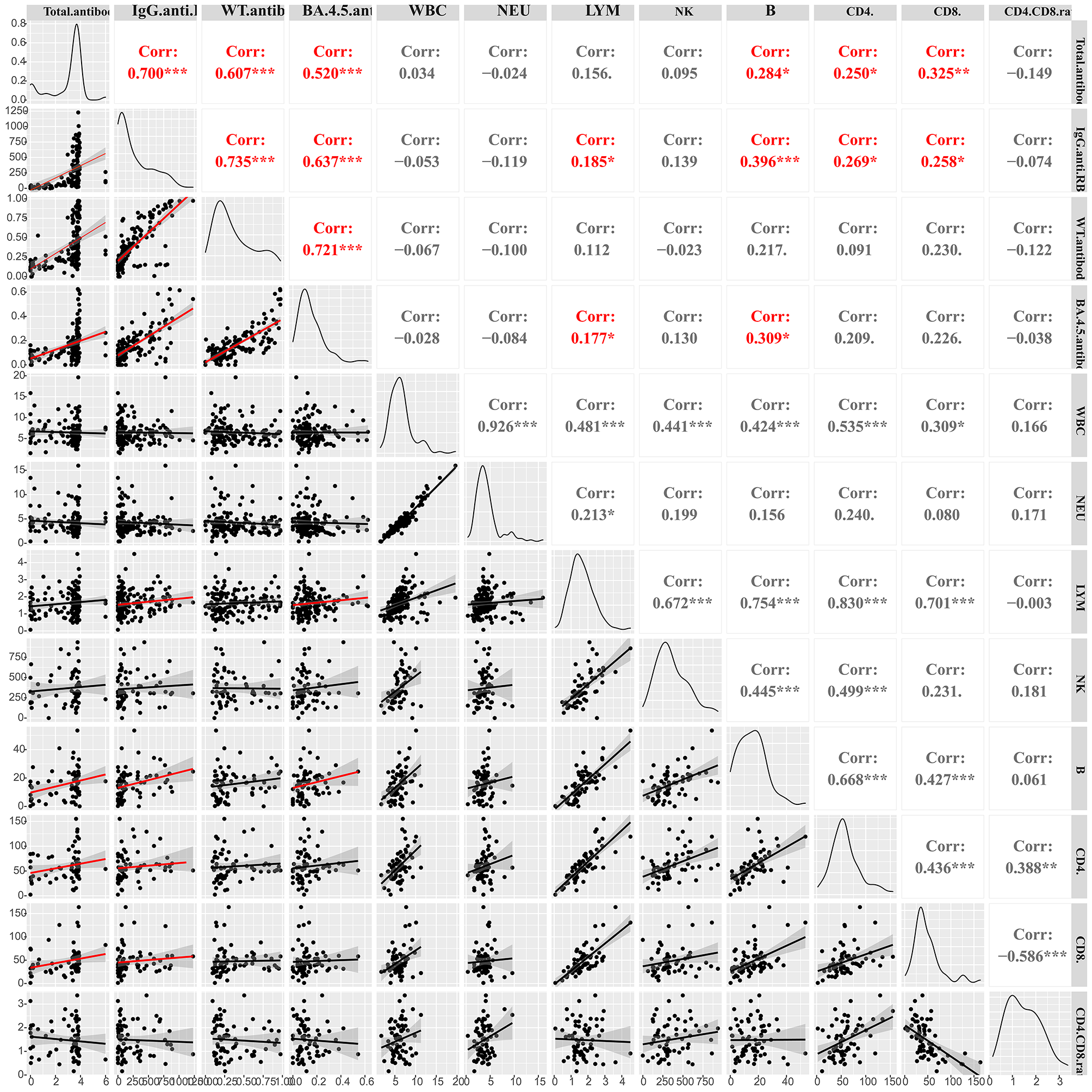

Supplement: Supplementary file 6 — Additional file 6: Figure S4. Correlation of biological variables and magnitude of SARS-CoV-2 antibodies after the booster dose. [file 13045_2023_1443_MOESM6_ESM.tif]
